# Tackling health literacy: adaptation of public hypertension educational materials for an Indo-Asian population in Canada

**DOI:** 10.1186/1471-2458-11-24

**Published:** 2011-01-11

**Authors:** Charlotte A Jones, Shefina Mawani, Kathryn M King, Selina Omar Allu, Megan Smith, Sailesh Mohan, Norman RC Campbell

**Affiliations:** 1 Faculty of Medicine, Departments of Medicine and Community Health Sciences University of Calgary, Libin Cardiovascular Institute of Alberta, Calgary, Alberta, Canada; 2 Department of Clinical Neurosciences, University of Calgary, Calgary, Alberta, Canada; 3 Faculty of Nursing and Department of Community Health Sciences, University of Calgary, Calgary, Alberta, Canada; 4 Hypertension Canada and University of Calgary, Calgary, Alberta, Canada

## Abstract

**Background:**

Indo-Asians in Canada are at increased risk for cardiovascular diseases. There is a need for cultural and language specific educational materials relating to this risk. During this project we developed and field tested the acceptability of a hypertension public education pamphlet tailored to fit the needs of an at risk local Indo-Asian population, in Calgary, Alberta, Canada.

**Methods:**

A community health board representing Calgary's Indo-Asian communities identified the culturally specific educational needs and language preferences of the local population. An adaptation of an existing English language Canadian Public Hypertension Recommendations pamphlet was created considering the literacy and translation challenges. The adapted pamphlet was translated into four Indo-Asian languages. The adapted pamphlets were disseminated as part of the initial educational component of a community-based culturally and language-sensitive cardiovascular risk factor screening and management program. Field testing of the materials was undertaken when participants returned for program follow-up seven to 12 months later.

**Results:**

Fifty-nine English-speaking participants evaluated and confirmed the concept validity of the English adapted version. 28 non-English speaking participants evaluated the Gujarati (N = 13) and Punjabi (N = 15) translated versions of the adapted pamphlets. All participants found the pamphlets acceptable and felt they had improved their understanding of hypertension.

**Conclusions:**

Involving the target community to identify health issues as well as help to create culturally, language and literacy sensitive health education materials ensures resources are highly acceptable to that community. Minor changes to the materials will be needed prior to formal testing of hypertension knowledge and health decision-making on a larger scale within this at risk community.

## Background

Cardiovascular diseases (CVD) are a leading cause of morbidity and mortality worldwide. Canadian data have suggested that Indo-Asians (including those whose ethnicities trace back to Afghanistan, Bangladesh, India, Myanmar, Nepal, Pakistan or Sri Lanka) have significantly higher rates of CVD morbidity and mortality compared with several other ethnicities [1,2]. Canadian migrant South Asians endure 2-5 fold higher rates of CVD morbidity and mortality compared with individuals of European descent [1-3]. The proportional mortality from ischemic heart disease (as a proportion of all-cause mortality) is higher in Canadian men and women of South Asian descent, (42% and 29%, respectively) as compared with men and women of European (29% and 19%) descent [2]. A more recent Canadian study found lower long-term mortality among South Asians compared with white patients with diabetes following acute myocardial infarction (AMI) [4]. However, AMI occurred at a younger age (average of 11 to 12 years) and recurrent AMI was 20% more likely in South Asians compared to white patients regardless of whether they had diabetes or not.

The higher prevalence of undetected and or inadequately managed risk factors including diabetes, central obesity, hypertension (HBP) and dyslipidemias, begin to explain the higher CVD risk profile [1,5,6]. Recent studies in the Indo-Asian community of Calgary Alberta [7-9] have determined that nearly one quarter of adults (>45 years of age) presenting for community-based screening have diabetes, 78% have dyslipidemias and 55% hypertension. Of significance, the control rates for hypertension and dyslipidemias in those screened were 50% lower than those reported for the general Canadian population [10]. There is clearly a need for culturally appropriate initiatives that address the prevention and control of such modifiable risk factors.

Indo- Asians are among the fastest growing minority populations in Canada. The 2006 Canadian census [11] determined that the self-identified Indo-Asian population rose by 37.7% from 2001 to represent one quarter of all visible minorities in Canada. Further, Indo-Asians comprise the largest and fastest growing cultural population in Calgary (only recently surpassing the rates for Chinese immigration) representing nearly 4% (approx 40,000) of Calgary's population (1,000,000+). Given such growth and the evidence that Canadian Indo-Asians are particularly susceptible to the CVD consequences of migration [1,2], it is of utmost importance to address access, language, and health literacy barriers in order to reduce this significant and disparate health risk.

Barriers to optimal health play a major role and include access, language and lower health literacy. Such obstacles may limit knowledge and appreciation of the ramifications of CVD and its modifiable risk factors [12-16]. Health literacy is 'the degree to which and individual can obtain, process, and understand the basic health information and services they need to make appropriate health decisions' [17,18]. Some have suggested that health literacy is at least as strong or stronger a predictor of health status than age, income, employment status, education level, self efficacy, race or ethnic group [19]. However, research on the relationship between literacy and health has been limited by the inability to separate out these complex factors that co vary with literacy. Enhancing health literacy, particularly of at-risk cultural minority groups, is an imperative step in improving health disparities.

This project represents the first step in a two-phase project aimed at increasing health literacy, hypertension knowledge, and health decision-making in an Indo-Asian population in Calgary, Alberta, Canada. This first phase was aimed at tailoring and evaluating the acceptability of adapted and translated versions of an existing English evidence-based public education pamphlet focused on hypertension.

## Methods

### Adapting the English hypertension education materials

Designed and updated annually by Blood Pressure Canada [20, http://www.hypertension.ca], the 2007 Public Hypertension Recommendations pamphlet uses evidence-based messaging to educate Anglo and Francophone Canadians about hypertension, its risk factors, prevention and treatment. It is a four page document split into three major sections. The first section, "Things you need to know about blood pressure and hypertension", describes what blood pressure and hypertension are, the cardiovascular consequences of poorly controlled hypertension and that hypertension can be prevented and controlled. The second section, "Do's and Don'ts" details the factors and activities (physical activity, weight loss if overweight, and a healthy diet) that promote a lower blood pressure and the factors (high sodium diet, excess alcohol, smoking) that promote higher blood pressure. Alongside these comments are colourful and simple pictures and green (Go) and red (Stop) signs. The third section, "Finding out about your blood pressure", provides information on where to get blood pressure measured, the purchase of a blood pressure device (machine), how to measure blood pressure at home and information on the need for and importance of medications and medication adherence in the treatment and control of hypertension. Pictograms inform the reader of what the numbers are for normal and high blood pressure, along with a sample of validated blood pressure devices that can be purchased in order to measure blood pressure at home.

The English pamphlet was tailored to the Indo-Asian audience using standardized principles for development of culturally, language and literacy appropriate patient educational materials [21-25]. Revisions of the materials were undertaken as dictated by the findings at each step of the procedure. The original English pamphlet was reviewed by a multi-disciplinary key informant (English speaking and reading) team consisting of four project team members, four lay individuals from the Indo-Asian community Health Board, two Indo-Asian health care providers from the local Regional Chronic Disease Management Program and two Indo-Asian lay volunteers known to the Health Board members. The team was asked to review the materials and identify potential barriers to the community members' understanding of the information contained within the pamphlet along with making suggestions to increase the cultural appropriateness and for the factors outlined in the 17-point checklist [22] and suitability of materials (SAM) [23] tools.

#### 1. Readability Assessment of the adapted English materials

The readability of the original (US grade-level 9) and adapted English (US grade-level six) pamphlets were confirmed using two different scales: manually, by the Fry method [26] and electronically, by the Microsoft Word^© ^2007 Flesch-Kincaid scale [27].

#### 2. Learner verification and revision

Five lay adult members of the Indo-Asian community (none of whom were part of the key informant team and each of whom were English speaking and reading) volunteered to review the adapted (grade-level six) pamphlet. Using the learner verification and revision [22] interview procedure (one on one with the project coordinator: SM) this team then verified the suitability, particularly the cultural appropriateness, of the health information.

#### 3. Translation of the adapted English material

The adapted English educational pamphlet was returned to the community members originally consulted, who once again reviewed it for cultural appropriateness, the 17 item check list and SAM prior to translation. Health Board members identified Gujarati and Punjabi as primary, and Hindi, and Dari as secondary languages of importance. Translations were done by Calgary Health Region Diversity Services [28] certified (experienced) translators with the exception of the Dari translation which was done by a local community resource. In order to verify the translation, the most common and highly recommended strategy of back translation [29] was used. Four members of the project team fluent in both English and at least one of Gujarati, Hindi, Punjabi, or Dari, back translated the four pamphlets to assure accuracy and concept (rather than literal) equivalence.

### Development of the evaluation tools

Two separate 14-item semi-structured evaluation tools (Tables 1 and 2) were developed in English by English and Indo-Asian research team members. The overall purpose of the tools was twofold: firstly, to assess the concept validity, and acceptability, and secondly to detect and revise mismatches in communication as well as unsuitable design and content of the adapted and translated materials. Questions, some of which were derived from the SAM, primarily addressed the participants' opinions on the attractiveness, graphics, layout, typography, cultural appropriateness. Additionally, the first tool (Table 1) was designed to be administered to English speaking participants who had read both the original and adapted English materials and included different questions with the major objective of comparing the two versions. The second tool (Table 2) was to be administered to non-English speaking participants who had read (or had the pamphlet read to them by a family member) one of the translated versions of the adapted pamphlet. The final question in each tool invited general comments about the pamphlets such that participants could express concerns or comments not addressed in the questionnaires.

**Table 1:** Adapted English version pamphlet questionnaire and responses.

Did you read the pamphlet?
Yes N = 59 No N = 3
**If not, Please explain why?**
• *"No time"*
• *"Didn't remember to read it"*
• No response

**Did you find the pamphlet helped you to understand what high blood pressure is?**
Yes = 56 (94.9%)* No = 3 (5.1%)

**Did you find the original pamphlet more difficult to understand compared to the adapted version?**
Yes = 8 (13.6) No = 51 (86.4%)

**Please tell us what part was difficult?**
• *"Prefer more pictures"*
• *"The original was very technical"*
• *"Not sure why"*

**Did you find the additional pictures in the adapted pamphlet helped you to understand the written information?**
Yes = 53 (89.8%) No = 6 (10.2%)

**Did you find the adapted pamphlet was missing information that you would have liked to see from the original?**
Yes = 3 (5.1%) No = 56 (94.9%)

**What information specifically was missing? (N = 3, one no response)**
• *"Low blood pressure was not discussed"*
• *"Do not know what"*

**If you could receive the material in a language other than English, what language would you choose?**
• N = 18 Gujarati ((30.5%), N = 3 Dari ((5.1%), N = 2 French ((3.4%), N = 1 Urdu (1.7%).

**Did you have any other comments about the pamphlet?**
• "*It was a good educational tool"*
• *"Well designed", Easy to read"*
• *"Well designed; simple to understand; gets you interested and started into understanding blood pressure specifically for a non-medical person"*
• *" Doctors do not have time to explain all this in their office"*
• *"Very good explanation of blood pressure"*

* Percents are based on the numbers of participants that read the pamphlet (N = 59)

**Table 2:** Translated version questionnaire and responses

Did you read the pamphlet?
Yes N = 30 No N = 5 No response N = 2
**If not, please explain why?**
• *Could not read N = 3*
• *"No time" N = 1*
• *"Just negligence" N = 1*

**Was it helpful to receive information on high blood pressure in your own language?**
Yes N =30 (100%)*

**Please explain why?**
No response N = 30 (100%)

**Did you find the pamphlet helped you to understand what high blood pressure is?**
Yes N = 30 (100%)

**Did the pictures help you to understand the information on high blood pressure?**
Yes N = 28 (93.3%) No N = 1 (3.3%) No response N =1 (3.3%).

**Which pictures were not helpful?**
*"Liked the vegetables picture but not the rest"*

**What part of the pamphlet did you like the best?**
*"All of it" N = 12 (40.0%)*
*"The Nutrition section" N = 5 (16.7%)*
*"The Do's and Don'ts Section" N =3 (10.0%)*
*"The exercise section" N =2 (6.7%)*
*"The written section" N =1 (3.3%)*
*"The pictures" N = 2 (6.7%)*
*No Response N = 5 (16.7%)*

**Did you have any other comments about the pamphlet?**
• "*Prefer a video in Punjabi, since I cannot read much (never gone to school)"*
• *"It will be more helpful if some information about blood cholesterol is also added since high blood pressure and adverse cholesterol levels influence cardio/stroke problem"*
• *"Medical University of Calgary started good program. It will be helpful to the community"*
• *"It is helpful for patients"*
• *"Provide information in Layman's terms"*
• *"It was very nicely done"*
• *"Satisfied with pamphlet"*
• *"Good"*
• *"No*" N = 7 (23.3%)
No response N = 6 (20.0%)

* Percent based on the number of participants that read the pamphlet (N = 30).

Each evaluation tool was assessed by a focus group composed of 5 volunteer members of the target Indo-Asian community who had read the pamphlets. The groups were instructed to review the questions in the tools to ensure that the themes of concept validity, acceptability, and comprehension in the pamphlets were clearly being assessed. Additionally, focus groups evaluated whether the questions being asked would be understood by other community members, particularly those whose preferred language was not English. Minor revisions were made accordingly.

### Distribution of the adapted and translated hypertension education materials

Three hundred and seventy five educational pamphlets were disseminated (265 English, 60 Gujarati, 34 Punjabi, 10 Dari, 4 Hindi and 2 data missing) to the local Indo-Asian population at 6 local religious and community centers as part of the educational component of a community-based culturally and language-sensitive CVD risk factor screening and management program (Indo-Central Asian-Cardiovascular Health and Management Program: ICA-CHAMP) [7]. Community and religious leaders invited participation in the program by public announcement at religious and community events. Self-selected participants were greeted in their native language and informed consent was obtained. Trained volunteers administered a CVD health questionnaire in the respondent's preferred language. Participants underwent a global CVD risk assessment [30] and brief risk counselling which included an explanation of the participant's risk factors and how they added up to predict their risk of CVD over the subsequent ten years. Participants then received one of the specially developed educational pamphlets in their language of choice and were asked to read them after the session. Participants were told they would be invited to evaluate the pamphlets when they returned for follow-up CHAMP sessions in one year. Participants fluent in English (Group 1) were given a copy of the adapted pamphlet in English to evaluate and compare with the original English pamphlet. Non-English speaking participants (Group 2) received a pamphlet in their native language of choice to evaluate at pre-arranged one year follow-up screening sessions.

### Evaluation of the educational materials

The evaluation tools were administered in the participants' language of choice by trained bilingual CHAMP volunteers at follow-up CHAMP sessions (seven to 12 months; mean 8.5 months after the initial sessions). The time between initial and follow-up screening sessions allowed for participants to visit their physician and for changes to risk factor management and lifestyle changes to be undertaken. Answers to the open ended questions were directly translated by the multi-lingual CHAMP volunteers and recorded in English. If program staff were not absolutely clear on the meaning of the words or phrases used by participants, extra time was taken to (probe) discuss and clarify the meanings.

Data from the closed-ended questions were presented as proportions. Qualitative responses to the open ended questions were transcribed verbatim and analysed by two of the investigators (SM, CAJ) using thematic content analysis [31]. Transcribed responses were analysed line by line and first level coding of common themes was performed by one investigator (SM). The other investigator (CAJ) then categorized, re-categorized and condensed some of the first-level codes based on overlap or similarity of some responses. Both investigators reviewed the second-level coding and generated consensus rules for inclusion within a code. Rules included participant descriptions that mirrored the pamphlet descriptions and those descriptions of hypertension documented in the literature [16] for a study on hypertension knowledge done in India. Participant responses were re-reviewed and coded independently by both investigators. Finally, independent coding was compared by the two investigators to obtain final consensus. Final coding results were tabulated, analyzed and presented as proportions in the same manner as the closed-ended questions.

This study was approved by the Conjoint Ethics Committee of the University of Calgary.

## Results

### Adaptation of the English hypertension education materials

After reviewing the original English pamphlet, the key informant team identified the following as potential barriers to the general community members' understanding: the inability of some community members to speak or read in English or their native language, the high volume of information presented in the pamphlet, the high technical level of information, and a lack of cultural appropriateness (particularly of the lifestyle advice provided). Using the 17- point checklist which assesses the organization, writing style, appearance and appeal of the materials and the SAM, which has been validated for English print materials in South East Asians [22], and scores 22 factors addressing the content, literacy demand, layout, typography, graphics, learning stimulation, motivation and cultural appropriateness the following changes were made to the original pamphlet: The font size was increased and total word count reduced by 25%. The total number of pages remained at four; however, the last page had no information on it and was reserved for the reader to make notes on. The language throughout the pamphlet was simplified using more basic words and phrases and words that could easily translate into other languages [21,23]. For example, the terms 'hypertension' and 'systolic' were changed to the more descriptive terms 'high blood pressure' and 'the top number'. Additional diagrams were added to reduce the amount of reading, improve understanding for lower literacy and non-English speaking groups [32] and to enhance the visual appearance of the pamphlet, for example a pictogram standard food label with a description how to read it and how to determine which foods have lower sodium "salt" content. A list of culturally appropriate food items to limit because of high salt content was added just below the food label pictogram (chutneys, chaat, massala, papad and namkeens).

Two other pictograms were inserted that demonstrate various blood pressure measurements and their significance (e.g. if you have diabetes, blood pressure greater than 130/80 mmHg, "your doctor will diagnose high blood pressure after two visits" or if your blood pressure is 200/120, "your doctor will diagnose high blood pressure right away"). In the do's and Don'ts section, tick marks were placed beside the "Do" actions as key informants indicated that some of the more elderly community members may not drive and recognize the significance of the green "go" or red "stop" signs. Some information, for example the section on "white coat and masked" hypertension and details about various anti-hypertensive medications was removed in order to reduce the excess of information.

### Readability assessment

Reading grade level of the material was assessed to be at the 6^th ^grade level [25,26].

### Learner verification and revision

No significant changes were made to the adapted English pamphlet prior to its being sent for translation.

### Translation of the adapted English material

The local Health authority was unable to provide translation for the Dari version of the pamphlet. After a national search for a certified translator failed to locate one, a local community member enlisted to provide this service. The certified and experienced translators provided a very literal translation of the materials. This proved to be suboptimal as our bilingual community members who performed the back translation thought that numerous words and phrases were inappropriate, not understandable or did not convey the real meaning for the less acculturated community members. A prime example was the direct translation of the words "processed foods" (in reference to hidden sources of high sodium content in the diet). According to the bilingual community members consulted, this did not hold a valid meaning for many non-bilingual Indo-Asian community members and "food sold in containers" would hold more meaning.

### Evaluation of the adapted and translated educational materials

Follow-up sessions were held in the same 6 venues as the initial screening sessions. Of the 375 participants originally screened, 248 qualified for a follow-up screening (age > 45, hypertension or one other risk factor) and 100 returned for repeat screening. Ninety-nine of the 100 participants that attended follow-up sessions agreed to participate in this study. There were no significant baseline demographic, risk factor or language preference differences between this group that returned for follow-up screening (and evaluated the materials) vs. the other 148 that qualified for recall screening but did not return for follow-up screening. 89/99 had read one or more of the pamphlets (90% attention). Group 1 (Table 1, N = 62) evaluated the English original and adapted pamphlets and Group 2 (Table 2, N = 37), who was non-English speaking, evaluated the translated versions of the adapted pamphlet. Respondents from group two (Table 3) were older and reported more diabetes than group one. 45.8% and 56.7% of Group 1 (English version) and Group 2 (Translated version) reported having being told they had hypertension or were on anti-hypertensive medications.

**Table 3:** Demographic and CVD risk factor profiles of participants who read and evaluated the materials.

Demographics	English Version (N = 59) N (%)	Translated Version (N = 30) N (%)

Female	26 (44)	16 (57)

Age (Mean in years [range])	60.6 (46-79)	69.1 (50-89) **±**

**Risk factors**		

Reported history coronary heart disease	4 (7.0)	2 (6.0)

Reported HBP or related medication use**	27 (45.8)	17 (56.7)

Reported dyslipidemia or related medication use ≠	34 (57.6)	13 (43.3)

Reported diabetes or related medication use	10 (5.9)	9 (30.0)

Reported family history of CVD	23 (39.0)	6 (20.0)

Measured BP above target+ at program entry	16 (27.1)	14 (46.6)

Measured cholesterol above target ++	39 (66.0)*	13 (43.3) ^†^

Identified as 'high risk'	38 (64.4)*	21 (70.0) ^†^

**± **Significantly (P < 0.05) older than Group one (Z-Test).* Cholesterol and risk level measurements not completed for 3 participants.^† ^Cholesterol and risk level measurements not completed for 5 participants. **, ≠ As diagnosed by a health care professional. + (>140/90 mmHg and 130/80 mmHg (in those with diabetes). ++ TC/HDL > 4 for high risk and > 6 for low/moderate risk participants.

Fifty-nine participants (Group one, Table 1) read both the original and adapted English pamphlets. Three stated they either forgot or did not have time to read the pamphlets. Thirty participants (Group two, Table 2) evaluated the translated versions (15 Punjabi, 13 Gujarati, 2 Dari). Seven did not read the translated pamphlet (four did not state a reason, three had no time or forgot and one stated "*could not read*"). No Hindi speaking participants attended the follow-up sessions.

### Group One, English Adapted Version

Fifty six of the 59 (94.9%) participants indicated that the adapted English pamphlet helped them better understand the meaning of high blood pressure than did the original English pamphlet.

Thirteen percent of participants indicated that the original pamphlet was more difficult to understand than the adapted pamphlet; however, only three responded as follows: (Table 1), *"Prefer more pictures", "The original was very technical" and "Not sure why"*. Interestingly, 53 (89.8%) of participants indicated that the increased number of pictures in the adapted version helped them to better understand the written information. Three participants felt that the adapted pamphlet was missing information (Table 1). One participant thought that low blood pressure should be discussed, one did not know what was missing and one did not respond. When asked if they would like to see the pamphlet in a language other than English, 40% said yes, (choosing Gujarati and Punjabi), 40% did not respond and the remainder said 'no'. None identified Hindi or Dari. Nine had additional comments: four indicated it was informative or very informative.

### Group Two, Translated Versions of Adapted English Version

All 30 participants who read a pamphlet indicated that it helped to receive the information in their own language (Table 2). None explained why. The majority of participants felt that the pamphlet had increased their understanding of hypertension. All respondents indicated that the information written in their own language and the pictures in particular (93%), helped improve their understanding of the meaning of high blood pressure.

When asked what part of the pamphlet they liked best and for any other comments about the pamphlet, the vast majority provided positive feedback. One participant would have preferred a "video in Punjabi", another would have liked information on cholesterol and one other wanted "information in laymen's terms". The sample size was inadequate to perform analyses by gender, age or language.

## Discussion

The goal of this community-based project was to adapt and determine the acceptability of an evidence-based hypertension educational pamphlet tailored to fit the needs of a local Indo-Asian population. Working with members of the target community to identify culturally specific educational needs, language preferences and to test the suitability of the materials allowed us in a five step process, to effectively adapt and translate existing evidence-based English language educational materials. Overall, participants found the educational tools highly acceptable. Participants felt the written and pictorial information was presented in a comprehensible fashion that helped them to better understand hypertension, its complications, treatment and prevention.

Development of these highly acceptable materials can be attributed to the active participation of and the strong partnership with the Indo-Asian community. Ninety-nine percent of the 100 CHAMP follow-up program participants agreed to evaluate and 90% had read the educational materials, suggesting a willingness to learn and play a role in program planning. Further, the target community was pivotal in securing the quality assurance of the materials at all steps of the development and evaluation.

Although self-selected, the 99 respondents had CVD risk factor profiles similar to those of the 248 participants that qualified for the initial CHAMP sessions, and to those reported in the literature [1]. The majority were at high risk for CVD with a high prevalence of uncontrolled risk factors. Despite this, 55/99 or 55% did not report having hypertension or related medication use and less than 10%, reported having experienced any CVD events. This mixed group represents an ideal target group for these educational materials whose objective is to promote improved awareness, increased prevention and management of their modifiable CVD risk factors.

Extensive literature and web-based search revealed that other than resources provided by the Heart and Stroke Foundation (http://www.heartandstroke.ca. Accessed October 12, 2009) dealing broadly with heart disease, stroke and their risk factors, these materials are unique as hypertension-specific educational materials that are appropriately translated for the Indo-Asian community. As such, these materials have the potential to address the awareness, prevention and treatment gap for hypertension in this high risk population.

There are several limitations to this study that need to be addressed by further research. First, initial CHAMP sessions provided an excellent forum to disseminate the adapted and translated Public Hypertension Recommendations. However, at the follow-up sessions, each participant took approximately 25 to 30 minutes to complete the ICA-CHAMP project procedures after which they completed the educational tool evaluation questionnaire. Time and the quantity of information relayed to the participants may have rendered them less attentive to the evaluation procedure. Additionally, the average 8.5 months between receiving and evaluating the materials may have affected recall and be responsible for those that did not provide a response or answered "Not sure why" to the question (Table 1), "Please tell us what part was difficult" or "Do not know what" to the question (Table 1), "What information specifically was missing?"; although participants made no mention of this being a problem.

Secondly, we did not evaluate the literacy skills of our participants prior to their evaluating the educational materials. Careful review of the literature revealed no validated methods of testing literacy levels in Indo-Asians. However, one study [33] piloted a brief literacy assessment in their British study of South Asians with diabetes. Some of the information collected in this assessment was obtained in our study; such as language preference, and reasons for not reading the materials. However, due to possible sensitivities around admitting the inability to read, this cannot be assumed to represent any form of literacy assessment. To compensate for this, we involved the target community in all steps of the development and evaluation of the materials. Reading grade level is a quantitative determination of reading difficulty and estimates the average grade level a person must have completed to be able to understand the (English) material. The goal for our materials was a grade level of six. There are no data on literacy skills of Indo-Canadians. However, there are data [34] suggesting that immigrants whose mother tongue is neither English nor French are almost three fold more likely to have low literacy skills (i.e. at the 6^th ^grade level or below). Furthermore, the Fry [26], and Flesch-Kincaid [27] are two accepted methods that consider a grade level of six or below as being superior for learners with low health literacy levels [22,24].

Coupled with the simplified pictures and diagrams, we were able to achieve a high degree of attention (interest in reading the materials) and acceptability in this population that clearly encompassed a broad range of literacy levels.

Thirdly, only three participants evaluated the Dari and none evaluated the Hindi versions, thus not allowing us to form any conclusions on these versions. Future interventions will strive to include more Hindi and Dari speaking participants in order to evaluate these educational materials.

Finally, because the participants in this small study (n = 100) were self-selected and not recruited in an epidemiological fashion, they are unlikely to fully represent the diversity within the Calgary Indo-Asian community itself. This may have lead to a bias favouring the characteristics of the particular groups represented herein, thus limiting the generalisability of study findings beyond this research setting. Additionally, this study design excluded those participants that cannot read. Larger, epidemiologically designed and controlled studies of health information delivery (e.g., audio-forms) need to be undertaken in order to include those who cannot read, those whose language has no written form [33] and those Indo-Asian sub-cultures not adequately represented herein. On the other hand, 66% of the sample read the English versions, which is consistent with the 2006 census data [11] indicating that 70% of Calgary's Indo-Asian community speaks English. This suggests that our study population may be somewhat representative of the large proportion of English speaking community members that may benefit from educational materials in English.

There is an abundance of literature guiding the development of health educational materials for low literate and multi-cultural populations. However, the vast majority of this research involves evaluating health educational materials that are written (or illustrations captioned) in English. Furthermore, there is a dearth of literature on health education research in hypertension for Indo-Asians. However, review of the most relevant literature reveals a consistent emphasis on the importance of cultural assessments, involving the target audience and the importance of accounting for low rates of literacy, even in the learner's native language [22,24,29,33].

The importance of involving our target audience in this health education and research was best exemplified in a British study of South Asians with diabetes [33]. The collection of questionnaire data dealing with diabetes self-management was found to be problematic because patients had poor literacy skills and failed to understand the meaning of the questions. The collection of valid data was compromised. To overcome this barrier they recruited individuals with diabetes from the community to participate in focus groups to successfully address and modify the content and mode of delivery of a self management questionnaire. In a similar fashion, Kai and Hedges [35] successfully used training and facilitation of resident community members to conduct semi-structured interviews within their own communities to inform the development of a service delivery model aimed at addressing a disparate ethnic health need.

Our five step iterative approach to the development and evaluation of the materials mirrored, in part that of Makosky and Daley [36]. For the cross-cultural adaptation of materials for Native Americans, they also used a modified SAM, in addition to two independent readability assessments along with target community input to successfully tailor and evaluate educational materials on smoking cessation in English. Specifically, they also found the SAM assessment inadequate for determining cultural appropriateness, suggesting that current validated tools may not be fully relevant or valid for non- English speaking people and used a learner verification method to modify their original untailored educational tools. Content experts undertook rigourous scientific review of their untailored materials while ours were originally developed by content experts. And finally, they had 2 independent individuals specially trained in the use of SAM review the materials, while we used a focus group format with a 12 member key informant team that was asked to review and comment on the materials guided by the factors outlined in the 17 point checklist and SAM tools.

We hired certified and experienced translators that unfortunately provided a very literal translation of the materials. This difficulty was addressed in our study by the process of back translation. This challenge was minimized in other studies [33,37,38] by the use of bilingual community members (with help from content experts) for the concurrent translation and adaptation of the original English source materials. Conceptual and cross-cultural equivalence of the materials was achieved in fewer steps and the process obviated the need for formal assessment of the original versus the adapted English versions.

The goal of this project was to tailor and evaluate the acceptability of the adapted and translated versions of an existing English version educational pamphlet. We did not formally evaluate the superiority of the adapted against the original English version. The adapted English version was designed following the recommendations of the 12 member key informant team. They had carefully reviewed the original version following the 17 point checklist of attributes and the SAM protocols along with making suggestions aimed at rendering it more culturally appropriate. Further, along with the readability assessment, and the informal assessment by the 56 English reading community members, the adapted version was considered at least equivalent to the original. Paradoxically, 90% of the individuals who originally stated that the original was no more difficult to understand than the adapted version stated that the pictures in the adapted version helped them to better understand the written information. This finding suggests at minimum, that the adapted version was equivalent or even superior to the original version. However, the question arises as to whether the original version even required adaptation for the English reading target audience. Further testing (below) will help to address this question.

All participants who read the translated versions of the pamphlet (Group 2) appreciated receiving the information in their language of preference and, like those in Group 1, found that the pictures helped to increase their understanding of the information. Further probing revealed that it was the additional pictures in the adapted version that assisted with a better understanding. This finding is supported by 2 review articles [39,40] showing that adding pictures to written (and spoken) health information can significantly improve patient attention (interest and acceptability), recall, comprehension and behaviours. They also present evidence suggesting that if pictures are appropriately simplified, they can be particularly helpful to those with low literacy skills.

Given the high level of acceptability of these materials, we believe the materials (original, versus adapted, and translated versions) are ready for formal randomized controlled testing among English and non-English literate ethnic community members to determine whether the adapted version is superior to the original version and to determine the effect upon change in hypertension knowledge and health decision-making of each version.

### Implications for further research and dissemination

A recent survey in Canada [34] suggested that immigrants whose mother tongue is neither English nor French are almost three fold more likely to have below basic (6^th ^grade level or below) literacy skills. Since the majority of patient health information is written at a 10^th ^grade or higher [24], a clear comprehension gap exists for much of the population. Tailoring health information to address lower health literacy skills can significantly increase acceptability, comprehension, recall and of particular importance, health behaviours [22,39,40] such as medication adherence.

The principles and methods of cross-cultural adaptation and translation of educational materials used in this study follow published guidelines [37,41] and have been validated in numerous other settings; for example, materials for smoking cessation in Native Americans [36], for obtaining informed consent in Indo-Asians [34] and for quality of life measures for Hispanic American patients [38]. The high level of acceptability of the materials within the target audience suggest that this process may be useful to other researchers who wish to develop targeted multicultural public educational materials for multiple purposes, within a broad range of multiethnic communities internationally.

It is imperative to involve the target community in identifying the key health issues as well as in the creation and review processes for health education materials [21,23,24]. In collaboration with the target community, we were able to modify the standardized/validated processes for creating and evaluating English language and translated materials to suit our target community. Scientific accuracy, reducing the complexity and achieving a 6^th ^grade readability level, simplifying the pictures and finally, ensuring cultural appropriateness of the educational materials are fundamental to improving health literacy and patient engagement. If individuals do not have the capacity to obtain, process and understand basic health information, they will not be able to make appropriate health decisions.

## Conclusions

Health literacy is fundamental to patient engagement and the potential for improved health outcomes. Involvement of the target community throughout the complete process of needs assessment through an evidence-based four step development and evaluation protocol is critical for the success and acceptability of culturally, language and literacy sensitive health education materials. This process may be useful to other researchers who wish to develop targeted multicultural public educational materials for multiple purposes, within a broad range of multiethnic communities internationally.

Improved community access to health education can be achieved utilizing trained local community volunteers at well frequented, geographically and culturally appropriate gathering places.

## Competing interests

The authors declare they have no competing interests. Funding agencies played no role in the design of the study or in any aspects of data collection, analysis, interpretation and reporting.

## Authors' contributions

CJ conceived of the study and along with SM, AOA, and MS made substantial contributions to conception and design, and acquisition, analysis and interpretation of data. CJ, SM, KK, and MS were involved in drafting the manuscript, and along with S Mahon and NC were involved with revising it critically for important intellectual content. All authors have given final approval of the version to be published.

## Pre-publication history

The pre-publication history for this paper can be accessed here:

http://www.biomedcentral.com/1471-2458/11/24/prepub
